# Subjective Experiences and Blood Parameter Changes in Individuals From Germany Following a Self-Conceived “Carnivore Diet”: An Explorative Study

**DOI:** 10.7759/cureus.82521

**Published:** 2025-04-18

**Authors:** Rainer J Klement, Johanna S Matzat

**Affiliations:** 1 Radiation Oncology, Leopoldina Hospital Schweinfurt, Schweinfurt, DEU; 2 Public Health, Society for Evolutionary Medicine and Health, Hamburg, DEU

**Keywords:** carnivore diet, cholesterol levels, hypercholesterolemia, ketogenic diet, low-carbohydrate diet, meat

## Abstract

Background: Animal-based, or so-called carnivore, diets largely exclude all plant-based foods and are gaining increasing popularity, mainly among individuals suffering from chronic diseases. This study aimed to explore subjective experiences and blood parameter changes of German followers of a carnivore diet.

Methodology: We conducted a statistical survey using a self-designed questionnaire and requesting blood panels. Inclusion criteria were: (i) following a carnivore-type diet for at least one month; (ii) completing the self-designed study questionnaire; and (iii) providing two sets of metabolic blood parameters from the period before and after adopting the carnivore diet. The survey was complemented by qualitative interviews with four subjects on a carnivore diet.

Results: Twenty-four individuals participated in the survey. Fifteen participants (62.5%) were male, and the median age was 46 (range 26-62) years. The majority (*n *= 16, 67%) reported at least one clinical diagnosis, and the main reason for switching to a carnivore diet was accordingly health-related. Improved health was also the major motivation to maintain the diet. Before the carnivore diet, participants consumed a variety of other diets, of which a ketogenic (*n* = 8) and standard diet (*n* = 7) were most frequently reported. There were no significant differences between on-diet and pre-diet blood parameters except for total (pre-diet median: 224 mg/dL; on-diet: 305 mg/dL; *P *< 0.0001) and low-density lipoprotein (LDL) cholesterol (pre-diet: 157 mg/dL; on-diet: 256 mg/dL; *P *= 0.00024) concentrations. However, two participants who initially had pre-diabetic HbA1c values and six participants with initially high (>130 mg/dL) triglyceride levels all experienced a reduction of these blood parameters during the carnivore diet.

Conclusions: Individuals adopting a carnivore diet do this mainly for health-related reasons and commonly experience subjective health improvements. Most blood parameters on the carnivore diet were within the reference ranges, and initially high HbA1c and triglyceride levels were reduced. However, the significant elevation of total and LDL cholesterol concentration is striking and warrants further investigation into potential adverse effects.

## Introduction

In early 1928, the Arctic explorers Vilhjámur Stefánsson and Karsten Andersen started a medically supervised, one-year-long exclusive meat diet to prove that such a diet consisting of muscle and organ meats, fat, and bone marrow is safe, which was confirmed by a team of physicians [1,2]. In 1975, Walter L. Voegtlin proposed in his book The Stone Age Diet that a meat-exclusive diet would best conform to the diet that humans evolved to eat during the Paleolithic era and hence to our physiology [3]. More recent data on human physiology and genetics, archaeology, paleontology, and zoology indeed provide evidence that at least since the occurrence of Homo erectus and lasting for many hundred thousand years, humans had occupied the highest position in the food chain, most likely classifying as hypercarnivores by the consumption of at least 70% animal matter [4].

Currently, there is a growing popularity of so-called *carnivore* or *zero-carb* diets, which are promoted partly by social media groups and can broadly be characterized by a strict focus on animal-sourced foods. Both terms are sometimes used interchangeably, and both belong to a spectrum of low-carbohydrate diets that also includes ketogenic diets, which are usually defined by high-fat (>70% of energy) and low-carbohydrate (<50 g/day) content. Two surveys aimed at identifying the motives for and benefits of adopting such diets in the longer term. Protogerou et al. obtained data from 170 international survey respondents who adhered to a *zero-carb* diet for at least six months and found that most of them were highly educated and maintained the diet due to improved health and well-being [5]. Lennerz et al. reached a sample size of 2029 survey respondents who were following a carnivore diet for a median of 14 months and confirmed that the prime motivation was health reasons, with 95% reporting improvements in overall health [6]. In addition to these two surveys, only a few case reports have been published in which a carnivore diet has been adopted either by a healthy individual [7] or by patients suffering from cancer [8,9], chronic inflammatory bowel disease [10,11], and other autoimmune conditions [12]. In addition, the survey by Lennerz et al. [6] has shown that individuals on a carnivore diet experience significant elevation in the low-density lipoprotein (LDL), high-density lipoprotein (HDL), and total cholesterol levels, a phenomenon that could be considered worrying. Hence, given the growing popularity of carnivore/zero-carb diets, there is comparatively sparse data regarding these diets’ effects and safety profile.

The main aim of this study was, therefore, to obtain further scientific knowledge about a carnivore diet on subjective and objective markers of health. Primary outcomes of interest were subjective changes in health status as well as pre-diet and on-diet blood parameters as objective markers of health, with a specific focus on blood lipid analyses. As secondary aims, we tried to evaluate the major motivational reasons why individuals adopt a carnivore diet, and to compare our findings to the US population of carnivores studied by Lennerz et al. [6].

## Materials and methods

This explorative study consisted of two parts. Participants for the first part were recruited in two consecutive years at the German Carnivore Convent, held on April 21-23, 2023, in Bad Hersfeld, and on April 12-14, 2024, in Bebra. At the convents, one of the authors (RJK) explained the main aims of the study and the inclusion criteria and provided his email address for individuals interested in participating. The inclusion criteria for the first part of the study were as follows: (i) consuming a carnivore-type diet for at least one month; (ii) completing the study questionnaire; and (iii) providing two sets of fasting blood panels, one representative of the pre-carnivore diet and one representative of the carnivore diet.

The study questionnaire was a quantitative questionnaire designed by one of the authors (RJK). It is shown in the Appendix and includes questions about individuals’ anthropometric measures, health issues, diet characteristics, and subjective changes on the carnivore diet. The questionnaire was partly constructed by adopting some of the questions used in the study of Lennerz et al. [6] and adding additional questions that asked about health-related aspects of participants. We took for granted that a carnivore diet is mostly composed of meat, eggs, and dairy, and therefore focused more on asking about non-animal-sourced food consumption. The questionnaires were handed out to participants in printed form or sent via email. In addition to answering the survey questions, we asked participants to deliver two fasting blood panels that had to come from certified medical laboratories in Germany. Parameters of interest focused on values which are frequently obtained in the context of medical checkups and included a small blood count panel, serum minerals (Na, K, Ca), serum glucose and HbA1c, serum lipids (total, LDL and HDL cholesterol, triglycerides), liver enzymes (AST, ALT, γ-GT), kidney function parameters (creatinine, uric acid), thyroid-stimulating hormone (TSH), and C-reactive protein (CRP).

The second part of the study consisted of collecting some qualitative data by conducting interviews with a small set of participants who followed a carnivore diet. The second author (JSM) conducted the interviews in August and September 2024. JSM is a social scientist experienced in qualitative research [13] and aware of the need for reflexivity [14]. She offers nutritional counselling and has academic and personal interest in a carnivore diet as a way to improve chronic disease conditions, without being on a carnivore diet herself. The four interviewees had previously received nutritional counselling from JSM on implementing a carnivore diet (three of them without financial compensation). One interviewee had also participated in the first part of the study. Inclusion criteria for the qualitative part were as follows:

(i) Consumption of a carnivore-type diet for at least one month

(ii) Willingness to tell one´s own story towards a carnivore-type diet

Briefly, the following questions were asked:

(1) “How did you hear about the carnivore diet? Why did you try it? Was there any key experience?”

(2) “Do you take nutritional supplements?”

(3) “What was your diet before you went carnivore?”

(4) “What was your motivation to start the carnivore diet? How long are you doing it?”

(5) “What did change after you switched to the carnivore diet? What has improved and what has worsened?

(6) “Do you plan to stay on the carnivore diet or switch to another diet?”

(7) “Do you see concrete obstacles to maintaining the diet in the long term?”

(8) “In your opinion, what are the pros and cons of a carnivore diet?”

(9) “Is there anything that we did not touch upon during the interview, but that you would like to mention?”

All quantitative data were pseudonymized and collated into an MS Excel® file and analyzed in R version 4.4.0. The pairwise Wilcoxon rank sum test was used to compare blood parameters before and during the carnivore diet. The chi-square test was used to compare the relative number of blood parameters outside the reference ranges before and during the carnivore diet. Given the explorative nature of this study, we did not correct *P*-values for multiple testing [15], but decided to use a more conservative threshold of *P *< 0.005 to claim statistical significance [16].

This study was conducted according to the Declaration of Helsinki. All participating individuals were fully informed about the purpose of the study, the requirements to participate, and the possibility to withdraw at any time. They provided their written informed consent to participate. After discussion within the first author’s institution (Leopoldina Hospital Schweinfurt, Schweinfurt, Germany) and consulting the ethics committee of the Bavarian Medical Association (email ID 2025-1051), the study was approved with no ethical concerns (most collected data were answers to a survey, and individuals had obtained blood parameters on their request and independently from this study).

## Results

General characteristics of participants in the statistical survey

**Figure 1:**
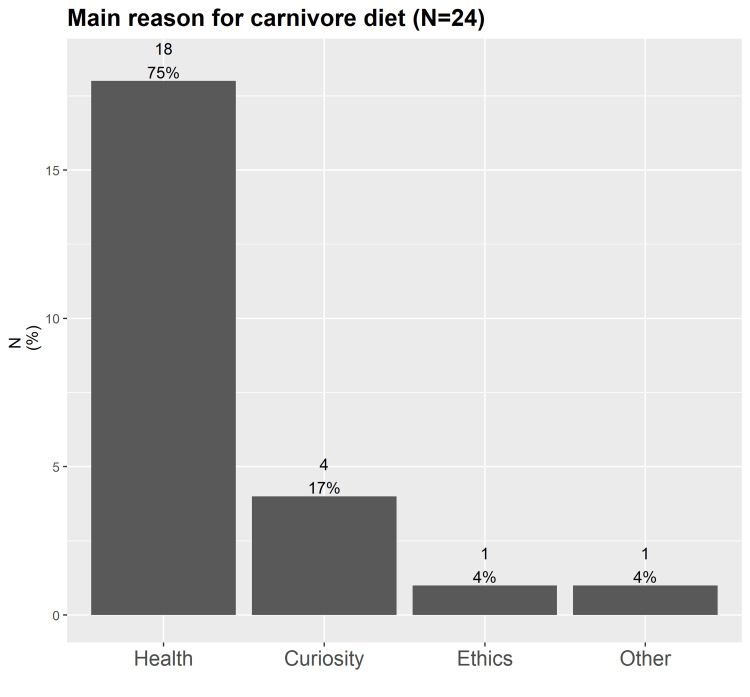
Distribution of the main reason for switching to a carnivore diet.

Twenty-four individuals volunteered to participate in the survey and provide pre- and post-carnivore diet blood parameters. Their baseline characteristics are given in Table 1. Most participants (15, 62.5%) were male, and the median age was 46 (range 26-62) years. Sixteen participants reported at least one clinical diagnosis; accordingly, the main reason for switching to a carnivore diet was health-related (Figure 1). Before the carnivore diet, participants consumed a variety of other diets, of which a ketogenic (*n *= 8) and standard diet (*n *= 7) were most frequently reported (a standard diet is any diet that is not consciously manipulated and/or does not belong to any of the other diet options in the questionnaire).

**Table 1:** Baseline characteristics of study participants. BMI, body mass index; IBS, irritable bowel syndrome; NAFLD, non-alcoholic fatty liver disease

ID	Sex	Age	BMI (kg/m^2^)	Smoker	Clinical diagnosis	Time on carnivore diet (months)	Meat consumption (g/day)	Exercise (h/week)	Previous diet
1	W	39	20.9	No	Polycystic ovary syndrome, burnout, depression, hypothyroidism	23	300	10-12	Paleo
2	M	34	21.8	Formerly	Acne, back pain, chronic fatigue syndrome, depression, hypotension	12	500	1-2	Standard
3	M	62	19.8	Yes	Arthrosis, hypothyroidism	6	250	1-2	Ketogenic
4	W	44	24.5	No	Prediabetes	5	400	0	Vegetarian
5	W	59	20.5	No	IBS	30	400	7-9	Standard
6	M	50	25.1	Yes	-	36	530	7-9	Standard
7	M	60	26.3	No	-	7	600	0	Paleo
8	W	45	20.9	No	Hashimoto-thyroiditis,	45	1000	3-6	Ketogenic
9	M	36	25.1	No	Ulcerative colitis	56	1200	>12	Standard
10	M	39	22.8	No	-	25	550	3-6	Ketogenic
11	M	53	21.5	No	-	3	900	3-6	Low carb
12	W	62	18.6	No	-	13	500	7-9	Primal
13	M	52	24.9	Formerly	Small-fiber neuropathy developed after COVID-19 vaccination, psoriasis, and NAFLD	3	500	1-2	Low carb
14	W	60	21.5	No	Polycystic kidney disease	16	400	1-2	Vegan
15	W	45	20.2	Yes	-	54	500	1-2	Ketogenic
16	M	27	17.8	No	Hashimoto-thyroiditis, IBS	17	850	3-6	Ketogenic
17	M	47	25.4	No	NAFLD, IBS, parasites, coagulopathy	7	400	7-9	Vegetarian
18	W	50	19.4	Formerly	-	1	500	7-9	Intermittent fasting
19	M	33	20.6	No	Hemorrhoids	18	900	3-6	Standard
20	W	44	25.2	Formerly	Adiposity	5	550	1-2	Ketogenic
21	M	34	28.7	No	Diabetes	4	800	7-9	Ketogenic
22	M	56	27.4	Formerly	Crohn’s disease	12	700	1-2	Standard
23	M	47	23.3	No	-	12	300	3-6	Intermittent fasting
24	M	26	24.4	No	Gingival recession, Mouches volantes	3	1,500	3-6	Ketogenic

Self-reported changes on the carnivore diet

**Figure 2:**
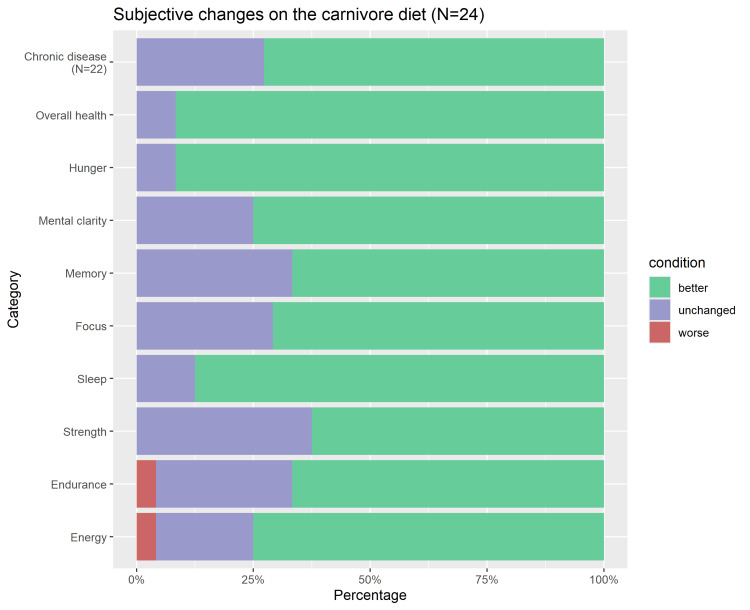
Subjective changes in health-related categories after switching to a carnivore diet.

The average duration on the carnivore diet was 17 months (median 12.0 months, range 1-56 months). The subjective changes experienced by individuals on the carnivore diet are displayed in Figure 2. The majority of participants reported improvements in all health-related categories. Only one subject each reported a worsening of energy levels and endurance.

Carnivore diet characteristics

According to the participants, the average self-reported daily meat intake was 626 g (median 515 g, range 250-1,500 g). Fifteen individuals (62.5%) characterized their diet as ketogenic, and four individuals (17%) as a raw carnivore diet. In descending order, the diets were composed of the following food items: organs in 22 cases (92%), eggs in 18 cases (75%), dairy products in 14 cases (58%), fish in 13 cases (54%), and honey in nine cases (37.5%). Of those consuming a ketogenic carnivore diet, four (27%) reported honey consumption.

**Figure 3:**
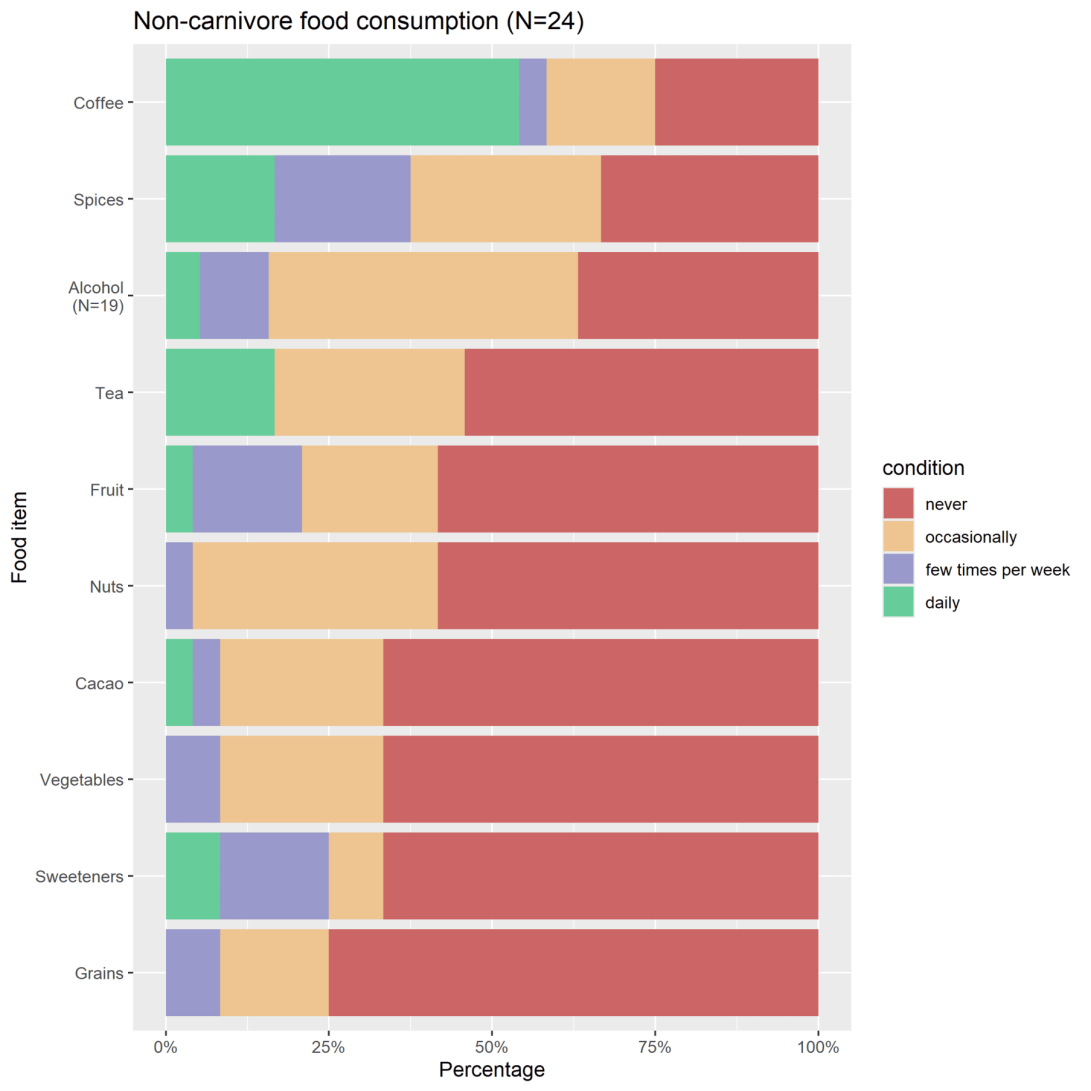
Frequency of consumption of non-carnivore food items.

The frequency of non-carnivore food consumption is plotted in Figure 3. The most frequently consumed non-animal-based food item was coffee: twelve participants reported daily and five others at least occasional coffee consumption. In contrast, the most avoided food item was grains, which were consumed occasionally by four or a few times per week by two individuals, respectively. Sweeteners, vegetables, and cacao were also generally avoided, while most individuals did at least occasionally include alcohol and spices in their diet. In total, six individuals (24%) completely avoided all solid plant-sourced foods and sweeteners; three of them never consumed any tea or coffee, two drank coffee daily or a few times per week, and one consumed tea occasionally.

Biochemical blood parameters

**Figure 4:**
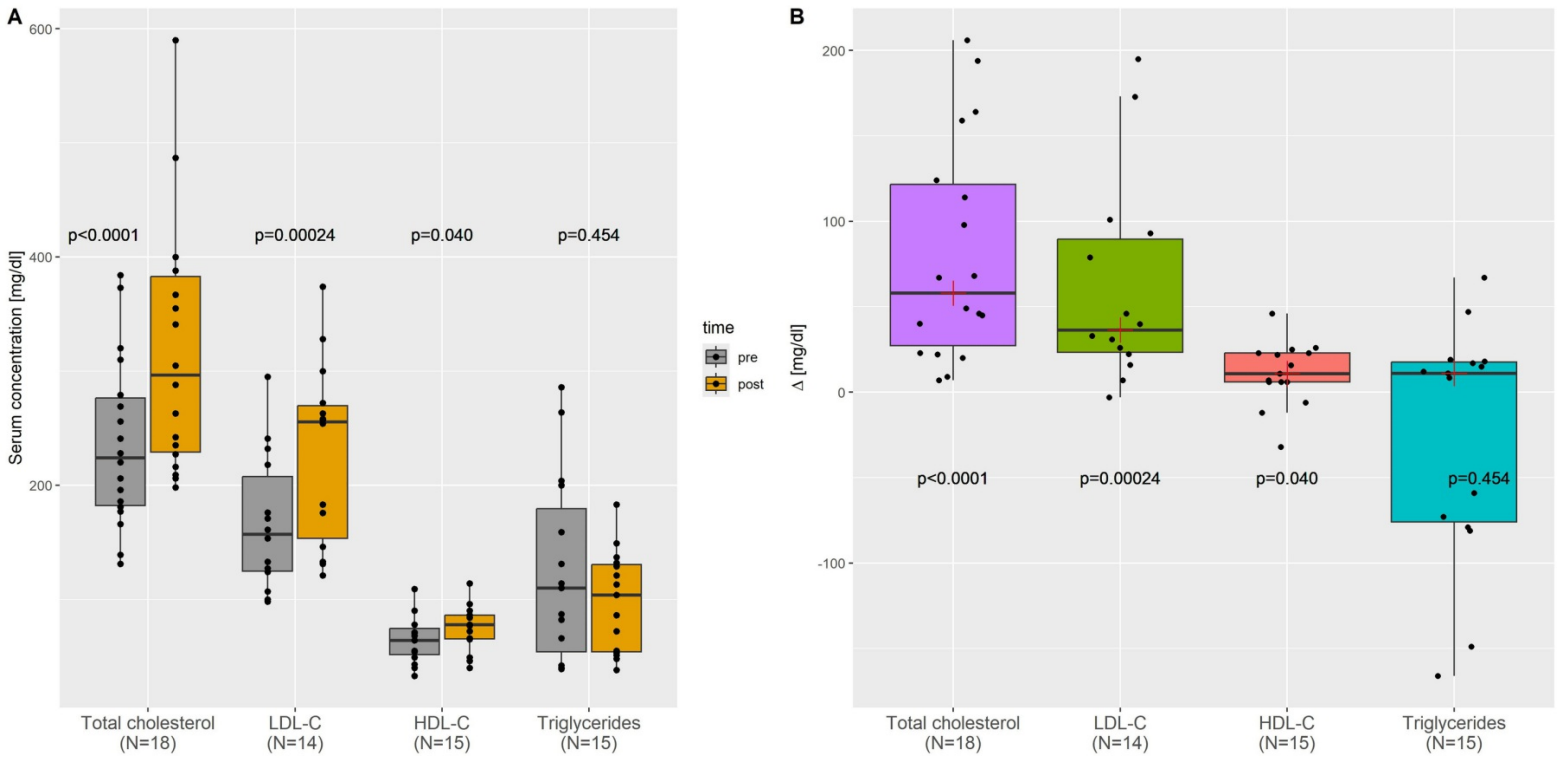
Changes in blood lipid parameters after switching to a carnivore diet. Left: Box plots showing the pre-diet and on-diet blood lipid parameters. Right: Box plots showing the median changes of blood lipid parameters. *P*-values correspond to the Wilcoxon matched-pairs signed-rank test.

The results of the laboratory blood analyses are shown in Table 2. On-diet blood parameters were obtained after a median of 11 months on the carnivore diet (range 1-54 months) and displayed no significant differences compared to the pre-diet values except for total and LDL cholesterol concentrations, which were raised significantly (Figure 4). Four participants initially had total cholesterol concentrations greater than 300 mg/dL; their characteristics are given in Table 3. Table 2 also contains reference values for the blood parameters considered. The thresholds for the triglyceride/HDL ratio were taken from Wakabayashi and Daimon [17], and for the LDL/HDL ratio from Sun et al. [18]. Although cholesterol levels were already high initially, they increased further after switching to the carnivore diet: total and LDL cholesterol exceeded their reference ranges in 7/18 (39%) and 7/14 (50%) of cases, respectively, for which pre-diet and on-diet values had been measured. Apart from cholesterol levels, pre-diet blood parameters were out of the reference range in 64/262 cases (24.4%), while this was reduced to 48/262 cases (18.3%) during the diet (chi-square test: *P *= 0.11).

**Table 2:** Pre-diet and on-diet comparison of blood parameters of interest. Differences between pre-diet and on-diet values were assessed for statistical significance using the Wilcoxon matched pairs signed rank test. *N*: number of participants for whom the specified blood parameters had been determined both before and during the carnivore diet; *V* (test statistic): sum of positive rank numbers. CRP, C-reactive protein; LDL, low-density lipoprotein; HDL, high-density lipoprotein; AST, aspartate aminotransferase; ALT, alanine aminotransferase

N	Parameter	Reference range	Pre-diet value	On-diet value	Change	*V* (test statistic)	*P*-value
20	Leukocytes (10^3^/μL)	4-10	5.5 (3.07, 21.9)	5.9 (3.75, 9.6)	0.07 (-14.6, 3.7)	96	0.984
19	Erythrocytes (10^9^/μL)	Males: 4.5-6.5; females: 3.9-5.6	4.70 (4.1, 5.8)	4.8 (4.21, 5.8)	0.06 (-0.52, 1.1)	66	0.408
19	Hemoglobin (g/dL)	Males: 14-18; females: 12-16	14.3 (12.9, 16.7)	14.7 (13.0, 17.2)	0.3 (-1.7, 2.9)	78.5	0.519
19	Hematocrit (%)	Males: 40-52; females: 12-16	43.2 (38.4, 50.8)	45.0 (39.2, 51.0)	0.9 (-4.4, 6.8)	69.5	0.500
20	Platelets (10^3^/ μL)	140-400	236 (132, 400)	229 (137, 365)	-10 (-112, 49)	117.5	0.376
18	Total cholesterol (mg/dL)	<250	224 (131, 384)	305 (198, 590)	58 (7, 206)	0	7.6 × 10^-6^
15	HDL cholesterol (mg/dL)	>60	64 (33, 109)	78 (40, 114)	11 (-32,46)	23.5	0.0404
14	LDL cholesterol (mg/dL)	<160	157 (98, 295)	256 (121, 374)	36 (-3,195)	1	0.00024
15	Triglycerides (mg/dL)	<150	110 (39, 286)	104 (38, 183)	11 (-166, 67)	74	0.454
14	Triglyceride/HDL ratio	Males: <2.967; females: <2.237	1.50 (0.44, 6.65)	1.45 (0.49, 2.80)	-0.27 (-3.86, 0.86)	80	0.0906
14	LDL/HDL ratio	<2.517	2.41 (1.26, 6.61)	3.08 (1.39, 6.45)	0.49 (-0.78, 1.54)	26	0.104
14	AST (U/l)	Females: <35	22 (15, 52)	20 (16, 27)	-1 (-33, 3)	66	0.161
16	ALT (U/L)	Females: <28	20 (11, 93)	22 (16, 47)	2 (-73, 19)	64	0.569
17	γ-GT (U/L)	Females: <40	18 (6, 60)	18 (11, 34)	0 (-36, 8)	93	0.760
10	Na (mmol/L)	135-145	139 (133, 142)	139 (137, 141)	0 (-2, 4)	16	0.797
11	Ca (mmol/L)	2-2.6	2.40 (2.16,2.51)	2.35 (2.28, 2.55)	0.06 (-0.19, 0.12)	22.5	0.374
12	K (mmol/L)	3-10	4.3 (3.2,5.35)	4.3 (3.9, 5.5)	-0.2 (-0.56, 1.2)	39.5	1
18	Creatinine (mg/dL)	Males: <1.3; females: <1.0	0.92 (0.6, 1.44)	0.99 (0.54, 1.3)	0.07, (-0.36, 0.35)	38	0.222
13	Uric acid (mg/dL)	Males: 3.6-8.2; females: 2.3-6.1	5.17 (1.78,7.2)	5.10 (3.4, 6.4)	0.3 (-1.42, 2.82)	29	0.273
9	CRP (mg/dL)	<0.5	0.60 (0.03, 2.8)	0.10 (0.03, 2.2)	0 (-2.7, 0.23)	13	0.178
11	Glucose (mg/dL)	70-110	85 (69, 117)	86 (77, 101)	1 (-37, 32)	26	0.721
8	HbA1c (%)	4.3-5.8	5.6 (5.0, 7.3)	5.5 (5.1, 6.6)	-0.1 (-0.7, 0.2)	25.5	0.324
9	TSH (μIU/mL)	0.35-4.5	1.41 (0.01, 2.13)	1.24 (0.02, 2.20)	-0.14 (-0.89, 0.86)	29	0.496

**Table 3:** Common themes that emerged from the qualitative interviews with concrete examples. A total of four participants were interviewed.

Common theme	Illustrative quotes
Naturalness	“And that was the point where I was caught: this naturalness ... Where it was said: if we all eat a vegan diet now, what will the landscape look like? Then it is a monoculture landscape. And then I thought to myself: that can't be right, it's not natural ... And it quickly became plausible for me, so I started eating meat again; and then I actually noticed the first time that I felt fully saturated again. I actually suppressed that a lot during the years when I went vegan … So for me it's actually through questioning: why are we here and how does the whole thing work? - So basically the topic of the order of creation and perhaps also the aspect: what is death? ... I see myself here as a human being, but also as a divine being who has a soul. And this soul also lives longer than the human being itself. That means, for me there is life even after death; and that's how I assume it is for animals and the like [...] and that the animals perhaps also have a certain function ... And if you eat meat, of course, you have to deal with the topic of death or killing, which is also one of those taboo topics in our society. And through this realization that there is life after death and reincarnation, I came to a different approach, namely that it is a natural process ... If you look at the natural world, there is always eating and being eaten. These are simply cycles, they are simply something natural that is part of the creation.” (P1, male, 39 years, ex-vegan)
	“I think what the government is doing right now with its dietary recommendations and attempts to get everyone to go vegan [...] and the recommendations to eat a maximum of 30 eggs in six months […] is very problematic.” (P4, female, 45 years)
Health	“I have been struggling with being very overweight for years. And when I heard from a friend that he had lost almost 20 kilos over the course of a few months using a carnivore-type diet, I thought to myself: I have to try that too.” (P2, male, 56 years)
	“The ultimate motivation to try carnivore was my autoimmune disease, because I've been trying everything possible to get rid of my psoriasis and get rid of the inflammation for over eight years; and nothing has worked, no matter how I ate ... And it just made sense to me … And I've had immediate success with it because my hairdresser sees it, he always asks me about it: ‘Ah, your skin is better today, so good. Your skin has never been like that.’ ... And I only ate like that for three weeks.” (P3, male, 38 years)
	“Because my Hb1Ac value kept getting higher and because I kept hearing from my alternative practitioner it was heading towards adult-onset diabetes ... And I wanted to avoid that at all costs … And then I thought to myself: I would like to get it under control and now I'm testing [the carnivore diet] for half a year, to see what it`s like and how it works … And that was my first sign that, on the one hand, the HbA1c had clearly decreased over time. And on the other hand, I had problems with my feet [...] and my foot problem, plantar fasciitis, disappeared within a week when I switched to a carnivore-type diet, i.e. by cutting out carbohydrates, and has never come back since. And those were actually the two key experiences, the long-term sugar level and the feet that kept me on the diet.” (P4, female, 45 years)
Simplicity and freedom	“I eat less than before, and I feel completely balanced, meaning I have enough energy. I have physical energy, I now train three or four times a week, and mentally, too, I feel like I can work with a lot of focus and concentration. Thinking back to when I was vegan, I often had midday slumps, which meant I wasn't able to work as concentrated as now.” (P1, male, 39 years, ex-vegan)
	“I just throw a steak in the pan and that's it. It couldn't be easier” (P2, male, 56 years)
	“The carnivorous diet is definitely a gain in freedom. I no longer have to write a shopping list. I go to my farm, get my 70-80 eggs for the week, and my meat from the butcher. And then it's fine; I no longer have to worry about what to buy, what to cook ... So the daily question: ‘What are we cooking today?’ is simply gone. So you simply feel much freer.” (P3, male, 38 years)
	“So if there's nothing suitable to buy or on the menu, I can bridge the gap by skipping a meal; that is, because the hunger isn't so pressing anymore. It's not so bad if there's nothing. And before, I thought I was going to die [...] not just in terms of energy levels, but also in terms of circulation, that typical hypoglycemic behavior.” (P4, female, 45 years)

**Figure 5:**
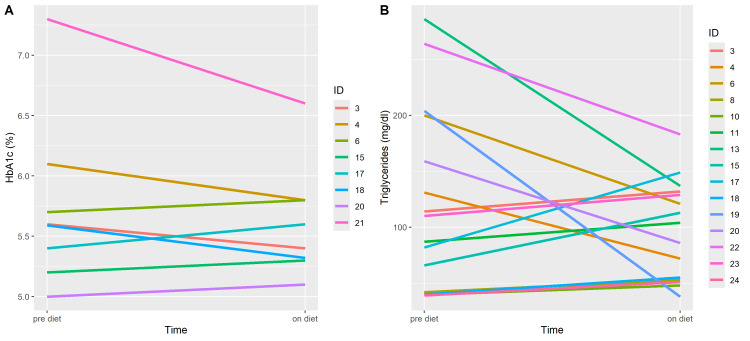
Individual changes in HbA1c values and triglyceride concentrations during the carnivore diet. The individual IDs correspond to the IDs given in Table 1.

Noteworthy, the individuals who initially had pre-diabetic HbA1c values and high triglyceride levels were able to lower these atherosclerotic risk markers; however, because some individuals with initially low HbA1c or triglyceride values experienced an increase in HbA1c or triglycerides, respectively, the overall difference between pre-diet and on-diet values was not statistically significant (Figure 5).

Reasons for switching to and maintaining a carnivore diet

Four individuals were interviewed for the qualitative part of this study; one of them had also taken part in the statistical survey. The mean duration of an interview was 30 minutes. In the qualitative interviews, three common themes emerged as motivations for starting or maintaining a carnivore diet, respectively: naturalness, health, and simplicity and freedom. Table 3 provides some quotes from our interviewees as representative examples for these common themes identified from the interviews.

Health reasons were reported as the main motivation for adopting a carnivore-type diet, similar to the results of the statistical survey. Several interviewees reported that before switching to a carnivore diet, they had suffered from chronic (e.g., plantar fasciitis) or autoimmune diseases (e.g., psoriasis, rosacea) for many years and that no therapeutic treatment had really helped them until then. Weight problems were another main motivation for most interview participants to switch to a carnivore-type diet, both underweight and overweight. What is striking is that all interviewees reported a quick, almost radical change after adopting a carnivore diet. Accordingly, most of the interview participants quickly experienced significant health improvements (e.g., reduced inflammation and pain, more beautiful skin), including improvements that they had not previously expected (e.g., less bloated stomach, no more feeling of fullness, better digestion, better satiety and more energy, more clarity, greatly improved sleep quality, better vision).

Another motivation mentioned by some interviewees is the desire to live in a closer connection to nature and therefore to eat more naturally again, i.e., to integrate more real food into one's own diet. Thereby, these interviewees also acknowledged that humans were mainly meat eaters during a significant period of their evolutionary history. And, finally, all participants pointed out the simplicity of the diet that subjectively gave them more freedom in their everyday life. Apart from the implicit acknowledgement that participants partly isolated themselves in terms of the social dining community of their family, they reported no subjective disadvantages of the carnivore diet.

## Discussion

This explorative study aimed to increase the knowledge about reasons why people choose to switch to a self-conceived carnivore diet and what some of the physiological effects are when doing so. The study was motivated by the increasing popularity of carnivore and animal-based diets, in particular among people with chronic diseases, and a previous study from the United States by Lennerz et al. [6], which produced interesting data about the motivation, characteristics, and health changes of individuals eating a carnivore diet.

Despite a significantly smaller number of participants, our results are qualitatively consistent with the main findings of Lennerz et al. [6]: First, the main reason why people adopted a carnivore diet was health-related. Second, all health-related aspects of quality of life improved in more than 50% of participants after they had switched to a carnivore diet, with specific proportions similar to those reported by Lennerz et al. (e.g., overall health: 92% versus 95% in Lennerz et al.; chronic disease 77% vs. 69%; mental clarity: 79% vs. 85%; hunger/food cravings 92% vs. 91%; endurance: 67% vs. 76%). Third, similar to the population studied by Lennerz et al., total, LDL, and HDL cholesterol increased markedly on the carnivore diet compared to pre-diet values, although we could not replicate the concurrent significant drop in triglycerides, HbA1c, and CRP concentrations reported by Lennerz et al. [6], possibly due to our small sample size. We also found that all other parameters besides cholesterol values tended to become more aligned to their reference ranges during the carnivore diet compared to pre-diet values (*P *= 0.11).

The subjective health improvements experienced by the majority of participants are in line with previous studies [5,6] and require an explanation. We propose three main mechanisms: One is that consumption of a carnivore diet supports a state of nutritional ketosis, i.e., an enhanced production of ketone bodies. Ketosis can exert pleiotropic health benefits mediated by the action of ketone bodies both as an energetic substrate and as signaling molecules [19,20]. For example, β-hydroxybutyrate, the ketone body with the highest concentration in humans, is an endogenous epigenetic modifier via histone acetylation and β-hydroxybutyrylation, thereby exhibiting anti-inflammatory effects [19]. The second mechanism is that the more or less complete elimination of plant foods concurrently eliminates plant chemicals that might be problematic for sensitive individuals. To avoid consumption by predators, plants produce so-called anti-nutrients, which can damage predators or make important parts of the plant inedible. During evolution, there was an arms race between plants inventing new anti-nutrients and predators responding by inventing new ways to detoxify those chemicals [21,22]. Contrary to the immune system, whose antibodies react specifically against particular antigens, animals evolved a detoxification system that can detoxify a broad range of chemicals. Hence, the same enzymes that evolved to detoxify plant chemicals nowadays protect us against anthropogenic environmental toxins. However, humans possess different detoxification enzyme polymorphisms, which lead to individual differences in the ability to detoxify particular chemicals [23]. We hypothesize that environmental toxins might tax the detoxification capacity of sensitive individuals to such an extent that the additional consumption of plant anti-nutrients results in the emergence of symptoms. The situation might be worsened by inflammatory processes, which can further inhibit the activity of detoxification enzymes [24]. In such individuals, the removal of anti-nutrients could relieve the detoxification system, leading to a subjective improvement of symptoms. Finally, a third mechanism for subjective improvements on a carnivore diet might be the high nutrient density of animal-based foods. Many micronutrients are more bioavailable or more or less exclusively available from animal-based foods; examples are choline, certain fatty acids (arachidonic, eicosapentaeic, docosahexaeic, and conjugated linoleic acid), taurine, carnosine, creatine, coenzyme Q10, and the vitamins A, B12, and D3. Consumption of an animal-based diet might, therefore, lead to rapid subjective improvements, especially in individuals who formerly consumed a vegan diet, as was testified by some of our interviewees. While these mechanisms appear plausible from a physiological perspective, they remain speculative because we have not measured ketone bodies, toxin loads, and micronutrient status of the participants.

Ketone body measurements would have been particularly interesting, given that the majority of participants (15, 62.5%) characterized their diet as ketogenic. Because a carnivore diet can contain significant amounts of protein as well as carbohydrates from dairy products, it is not necessarily a ketogenic diet, although the term is sometimes used interchangeably with the term *zero-carb diet*. According to Protogerou et al., the term *zero-carb diet* does not refer to a specific diet with a fixed macronutrient ratio or set of allowed foods, but rather to a range of low-carbohydrate diet variants that occupy the extreme end of a continuum of other low-carbohydrate diets, all characterized by food choices predominantly consisting of animal-sourced foods [5]. However, this is contrary to how some of the first practitioners of a *zero-carb diet* define this term:

“A Zero Carb diet is the ultimate ketogenic diet […] The Zero Carb Diet as we defined it, is a diet of animal products only” [25].

We also propose defining a zero-carb diet as a variant of the carnivore diet, consisting exclusively of meat, eggs, butter, and cheese, which contain only trace amounts of carbohydrates, whereas a carnivore diet may include a certain percentage of complementary plant-sourced foods. The human carnivore diet, as defined here, is analogous to the category of hypercarnivores described by Van Valkenburgh [26], referring to mammals that consume more than 70% of their diet as vertebrate-derived matter (e.g., the domestic cat, Felis catus, an obligate carnivore, consumes a diet comprising over 90% animal-sourced foods [27]). Using these definitions, five of our survey participants (20.1%) followed a zero-carb diet that excluded all solid plant foods (such as fruits, vegetables, nuts, chocolate/cacao, and grains) as well as sweeteners, while the remaining participants adhered to a less stringent carnivore diet that included occasional plant-sourced foods. Future studies should collect detailed data from food diaries or food frequency questionnaires to determine the maximal threshold of plant-sourced foods that adherents of a carnivore diet typically eat.

It is currently unclear if a long-term carnivore diet increases the risk of cardiovascular disease. Epidemiological studies have found significant positive associations between the intake of meat and an increased risk of cardiovascular disease [28], while the intake of fiber is associated with a better cardiovascular disease risk profile [29]. Although it has not been established that such associations are causal [30,31], the food composition of a carnivore diet and the significant increase in total and LDL cholesterol levels experienced by adherents to this diet may be reason for concern. Data from randomized controlled trials indicate that carbohydrate restriction to <40% energy intake typically lowers triglyceride and elevates HDL cholesterol concentrations, while the effect on LDL cholesterol concentrations is more variable [32]. However, if the randomized trials are restricted to normal-weight adults under severe carbohydrate restriction of <10% energy intake - which is more representative of our study population - the typical result is a significant elevation of both LDL and total cholesterol levels [33].

The research group of Nicholas G. Norwitz, David Feldman and Adrian Soto-Mota conducted a meta-analysis of randomized trials and showed that the propensity for elevations in total and LDL cholesterol concentrations increases as the BMI of individuals undertaking a low carbohydrate diet decreases [34]. In other words, leaner individuals are more likely to experience an increase in their LDL and total cholesterol levels when they adopt a very low carbohydrate diet. This meta-analysis confirmed prior findings from the same group, which had conducted an online survey of 548 adults consuming a low-carbohydrate diet - both lower BMI and a smaller triglyceride/HDL ratio, a maker of good metabolic health, was associated with a greater increase of LDL cholesterol levels [35]. A subset of individuals with LDL cholesterol ≥200 mg/dL, HDL cholesterol ≥80 mg/dL and triglycerides ≤ 70 mg/dL was defined as having a *lean mass hyper-responder* (LMHR) phenotype, with some individuals exhibiting LDL cholesterol levels above 500 mg/dL, values that are otherwise only observed in patients with familial hypercholesterolemia (FH) [35]. Norwitz et al. went on to offer a theory - the lipid energy model - according to which individuals on low-carbohydrate diets respond to low insulin levels and decreased liver glycogen by increased release of non-esterified fatty acids from adipose tissue, which serve as a fuel for oxidative tissues such as skeletal muscle [36]. The liver also takes up non-esterified fatty acids and repackages them as triglycerides into VLDL particles. These VLDL particles are sent to peripheral tissues where they get *unpacked* through the action of lipoprotein lipase. While the triglycerides get broken down again into non-esterified fatty acids and then taken up into cells, the VLDL particles *shrink* to LDL particles, in the process losing some surface remnants such as cholesterol, phospholipids, and apolipoproteins, which are taken up by HDL particles. Thus, increased VLDL particle secretion would result in both an increased LDL and HDL particle mass and cholesterol content [36]. This is expected to occur to a greater extent with greater restriction of carbohydrates, lower fat mass, and higher energy demands such as through exercise.

The lipid energy model also explains our data. There was a significant increase in LDL and total, and to a lesser extent HDL cholesterol in this non-obese population after adopting a carnivore diet (Table 2). Two individuals also classified as LMHRs, having LDL and HDL cholesterol levels greater than 200 and 80 mg/dL, respectively, with concurrently low triglyceride levels below 70 mg/dL. Their BMIs were 21.5 and 19.4 kg/m^2^, respectively, and they exercised for 1-2 and 7-9 hours per week (IDs 14 and 19 in Table 1). The health implications of the increased cholesterol levels in our cohort, and the two LMHRs in particular, are currently the subject of scientific debate [37,38]. Although LDL cholesterol levels of LMHRs on a low-carbohydrate diet are comparable to those of patients with FH, there are important distinctions. First, FH is an inherited condition, most often caused by autosomal dominant mutations in the LDL receptor gene, leading to elevated LDL cholesterol levels from birth [39], whereas LMHRs lack such mutations and exhibit increased LDL cholesterol levels only during periods of carbohydrate restriction. Second, it is a main prediction of the lipid energy model that increased carbohydrate consumption reverses the rise of LDL cholesterol in LMHRs, which has meanwhile been confirmed experimentally [40]. In contrast, the effect of diet on LDL concentrations in FH is only modest, even with very low saturated fat and cholesterol intakes [39]. Third, triglyceride and HDL cholesterol levels are, by definition, ≤70 and ≥80 mg/dL, respectively, in LMHRs on low-carbohydrate diets, while triglycerides are typically >100 mg/dL in patients with FH and HDL cholesterol levels can be quite low. Low HDL cholesterol and high triglycerides are also much better predictors of cardiovascular disease risk than LDL cholesterol levels in FH patients [41]. Fourth, FH patients typically develop dermatological signs of cholesterol depositions in the form of xanthomas [39], which so far has not been reported for LMHRs [38].

It is thus unclear to what extent LMHRs, which are metabolically healthy from every aspect except their high LDL cholesterol levels on low-carbohydrate diets, resemble FH patients with respect to cardiovascular disease risk. The KETO-CTA (Ketogenic Diet and Coronary CT Angiography) trial tested whether increased LDL cholesterol levels would translate into increased coronary artery calcium (CAC) scores, a major predictor of atherosclerosis and coronary events [42]. In the first phase of this trial, the investigators compared 80 LMHRs, who had followed a low-carbohydrate diet for 4.7 ± 2.8 years and displayed total and LDL cholesterol levels of 369 ± 95 and 272 ± 91 mg/dL, respectively, with 80 matched individuals from the Miami Heart study who had total and LDL cholesterol levels of 205 ± 40 and 123 ± 38 mg/dL, respectively. The median CAC scores were 0 in the low-carbohydrate group and 1 in the Miami Heart study group, which was not significantly different (*P *= 0.520). In addition, no significant association between LDL cholesterol and CAC scores was found. In the recently published second phase, the authors had prospectively followed 100 LMHRs on a low-carbohydrate diet for one year and found no associations between high LDL cholesterol exposure time, baseline apolipoprotein B, or change in apolipoprotein B levels and plaque progression; instead, baseline CAC score was a significant positive predictor of plaque progression [43]. The results of the KETO-CTA trial were consistent with data from other clinical trials and FH research, indicating that LDL cholesterol levels are poor predictors of cardiovascular disease risk in otherwise metabolically healthy individuals [42]. Nevertheless, further research with more long-time follow-up data is necessary to clarify any remaining potential risks associated with the LMHR phenotype in the context of a long-term carnivore or other low-carbohydrate diet.

Our study has several limitations that might bias our results or decrease their external validity. First, we only had a small sample size, which could have hampered our ability to detect less pronounced blood parameter changes. However, despite the small sample size, we were able to qualitatively reproduce many results obtained in the much larger study by Lennerz et al. [6] and detect significant blood parameter changes in cholesterol levels. Second, the duration of the diets and the time points at which participants reported their data varied widely, which could introduce bias in the pre-diet versus on-diet comparisons and reduce the external validity of our results. Third, most data were self-reported and, therefore, unverified by an external examiner such as a physician. However, we could at least confirm many of the self-reported subjective changes by direct interviews with four individuals. Furthermore, contrary to the study by Lennerz et al. [6], the blood parameters of our participants were not self-reported but taken from scanned documents coming from certified medical laboratories. Fourth, as also pointed out by Kirwan et al. [44], our study participants might belong to a very specific group of people that is differentiated from the general population by a multitude of health behaviors of which only a small subset was included in our survey. This also makes generalized inferences from this study more difficult. Fifth, we did accurately measure dietary intakes, so, as mentioned previously, it remains to be determined what exactly constitutes a carnivore diet. Finally, a major limitation concerns the lack of a control group against which subjective and objective markers of health could have been compared. Thus, our findings should be interpreted as hypothesis-generating and should be confirmed or refuted by future controlled trials.

Despite these limitations, our data are useful as they provide the very first broad characterizations of the health status, motivation and behavior of German individuals following a carnivore diet. Given the growing popularity of carnivore or animal-based diets, future studies, including interventional trials, should be undertaken to further investigate the metabolic and subjective changes that occur when individuals adopt such diets. Lastly, the health implications of the carnivore diet especially for individuals who follow this diet for longer periods or who possess a lean mass hyper-responder phenotype remains to be evaluated.

## Conclusions

Using a quantitative survey of 24 individuals and four qualitative interviews, we studied a sample of followers of a carnivore diet from Germany to investigate their motivation and subjective experiences on this diet, as well as changes in important blood parameters. Despite several limitations, especially the small sample size and self-reporting of outcomes, our study provides valuable insights into the motivation and experiences of individuals following a carnivore diet. We found that the main reason why people adopted a carnivore diet was health-related, mostly due to chronic (in particular autoimmune) diseases and overweight. The health-related aspects of quality of life that we had assessed improved in the majority of participants after they had switched their diet. This was particularly pronounced concerning overall health. Except for LDL and total cholesterol concentrations, the overall blood count also improved on the carnivore diet, although this was not statistically significant. However, total and LDL cholesterol concentrations increased significantly on the carnivore diet, which is a reason for concern and warrants further investigations.

To conclude, the study provides preliminary evidence that most blood parameters remain more or less unaffected by adopting a carnivore diet, but that total and LDL cholesterol levels can increase to an extent that appears worrying. With the recent discovery of a so-called LMHR phenotype of a small fraction of individuals who experience significant elevations of cholesterol levels on a low-carbohydrate diet, future studies should investigate the physiology behind this phenomenon and study any putative pathological consequences.

## Appendices

Appendix: Carnivore study questionnaire

Name: …………………………………………………………………………………………...

Age: ………… years

Sex:     □ male             □ female

Height: …………cm              Body mass: ………… kg

Smoker:          □ yes (currently)         □ yes (formerly)                     □no

Clinical diagnoses ascertained by a physician or naturopath:

……………………………………………………………………………………………………………………………………………………………………………………………………………………………………………………………………………………………………….

1. How long have you been following the carnivore diet in its current form?

………… months

2. For what main reason did you change your diet to carnivore? (only one answer)

□ To improve my health/optimize my body weight

□ For ethical reasons

□ Out of curiosity

□ Because of the choice of food/ for reasons of taste

□ Other reasons: ………………………………………………….

3. For what health reasons did you switch to Carnivore? (Multiple answers possible)

□ To reduce body weight

□ To optimize body composition

□ To improve “brain fog” and have more energy

□ To get type 2 diabetes under control

□ To stabilize blood sugar in type 1 diabetes

□ To improve osteoarthritis / inflammation / joint pain

□ To improve mental problems/ depression/ anxiety disorders/ burnout

□ To get an autoimmune disease under control

□ To get digestive problems under control

□ To get allergies/skin problems under control

□ Others: …………………………………………………………………

4. Which of the following diets best describes your diet before switching to carnivore? (only one answer)

□ „Balanced“  □ Vegan          □ Vegetarian               □ LOGI/Low Carb     

□ Ketogenic                □ regular intermittent fasting

□ Paleo                □ Other ..........................................................................................

5. Which of the following characterizations can be used to describe your carnivore diet? (multiple answers possible)

□ Raw Carnivore                    □ Ketogenic

□ With dairy products            □ Without dairy products except for butter    

□ With offal                           □ With fish        

□ With honey                         □ With eggs

6. How many grams of meat + offal do you eat on an average day?

……….. gram

7. How often do you eat the following non-carnivorous foods?

Coffee                □ daily             □ a few times a week             □ even rarer    □ never

Tea                      □ daily             □ a few times a week             □ even rarer    □ never

Vegetables         □ daily             □ a few times a week             □ even rarer    □ never

Fruits                  □ daily             □ a few times a week             □ even rarer    □ never

Grains                 □ daily             □ a few times a week             □ even rarer    □ never

Nuts/ seeds        □ daily             □ a few times a week             □ even rarer    □ never

Cacao                 □ daily             □ a few times a week             □ even rarer    □ never

Spices                 □ daily             □ a few times a week             □ even rarer    □ never

Sweeteners         □ daily             □ a few times a week             □ even rarer    □ never

Alcohol                □ daily             □ a few times a week             □ even rarer    □ never

8. How often do you take food supplements?

Vitamins                         □ daily             □ a few times a week □ even rarer    □ never

Minerals                          □ daily             □ a few times a week □ even rarer    □ never

Ω-3 fatty acids               □ daily             □ a few times a week □ even rarer    □ never

Protein/ amino acids      □ daily             □ a few times a week □ even rarer    □ never

Others                             □ daily             □ a few times a week □ even rarer    □ never

What exactly do you take as a dietary supplement?

……………………………………………………………………………………………………………………………………………………………………………………

9. Within the last 3 months, how would you rate the following criteria compared to the time before you started the carnivore diet?

Overall health                □ Better          □Unchanged               □Worse

Energy level                   □ Better          □Unchanged               □Worse

Strength                         □ Better          □Unchanged               □Worse

Endurance                      □ Better          □Unchanged               □Worse

Focus                              □ Better          □Unchanged               □Worse

Mental clarity                □ Better          □Unchanged               □Worse

Memory                          □ Better          □Unchanged               □Worse

Sleep                              □ Better          □Unchanged               □Worse

Chronic Diseases           □ Better          □Unchanged               □Worse

Hunger/ cravings            □ Better          □Unchanged               □Worse

10. How do you find implementing the carnivore diet in everyday life?

□ Very easy    □ Easy             □ Neutral        □ Difficult      □ Very difficult

11. Are you currently taking medication?

□ No               □ Yes (please specify): ……………………………………………………. …………………………………………………………………………………………...

12. How many hours of sport do you do on average per week?

□ No exercise         □ 1-2 h     □ 3-6 h         □ 7-9 h       □ 10-12 h       □ > 12 h

13. Which sport(s)? ……………………………………………………………………………

……………………………………………………………………………………………..
